# How Does a Healthy Interactive Environment Sustain Foreign Language Development? An Ecocontextualized Approach

**DOI:** 10.3390/ijerph191610342

**Published:** 2022-08-19

**Authors:** Hao-Zhang Xiao

**Affiliations:** School of Foreign Studies, South China Agricultural University, Guangzhou 510642, China; sshaw@scau.edu.cn

**Keywords:** sustainable learning, sound-meaning mapping prioritizing route, ecocontextualized approach, tiered learning interactive context, sequenced implicit-explicit awareness, role awareness

## Abstract

Recent inconsistent empirical findings on the impact of context on foreign language development (FLD) are related to some conflicting context views, which hinders healthy FLD. Given this, an ecocontextualized approach/perspective is presented as a ”recipe”, holding that inter-intrastratally interactive context-oriented learning starts with the alignment of implicit sound-meaning mapping (phonic listening and speaking only) with the low cognitive levels of early starters and physical objects/visual realia in the proximate context, and only when the learners’ cognitive levels develop several years later can it turn to explicit formal learning through abstract written language and contents. Based on this view, a sound-meaning mapping prioritizing (SMMP) route to healthy/sustainable FLD is proposed and testified via questionnaires and an interview/oral test. Results showed: (1) SMMP early starters surpassed the non-SMMP (NSMMP) early starters (learning reading, listening, speaking, and writing simultaneously) in oral proficiency at the late stage despite their homogeneity at the early stage; (2) oral, especially listening abilities, could not be well developed at the late stage by NSMMP learners; (3) written proficiency could be developed later by both types. These findings reveal the SMMP route to healthy/sustainable FLD in the Chinese context.

## 1. Introduction

The role of environment/context in language learning has attracted increasing interest [1,2,3]. However, the concept of context and its underlying learning views vary among researchers and remain vague given their paradigm shifts from the socio/linguistic [4,5,6], to the cognitive/innatist [7,8], and to the sociocognitive/sociocultural [9,10,11,12] schools. These views diverged from their inceptions, lacking communication and coordination. Contextually, the late cognitive context models obfuscate the demarcation between the traditional contextual factors [13]. As a result, increasing arguments and incompatible findings emerge in second language acquisition (SLA) or FLD studies. For instance, empirical studies have focused on partial or indistinct context effects during a period of time or at a time such as study abroad vs. domestic classroom context effects [14,15,16] or study abroad university vs. au pair settings [2] or study abroad vs. domestic international community settings [17] or classroom vs. laboratory settings [1,18,19] or dis/similarities between early and late foreign language learning effects [20,21]. 

Such differences in either contextual concepts or empirical findings have been at large for ages in SLA, lacking a consideration of contextual order parameters across timescales and ranges within a dynamic and holistic contextual framework. Given such a research lacuna, this study aims to present an ecocontextualized approach/perspective with a focus on the interactions between language(s) and environments/contexts, viz. an ecointeractive context-oriented perspective that coordinates external (sociocultural, natural/physical), linguistic (phonic, multimodal, graphic), and internal (cognitive, psychological, neuro/genetic) parameters through sequential inter-intrastratal interactions. From this perspective, it will then investigate a route for healthy/sustainable FLD regarding early learning effects that will be verified by structured questionnaire surveys and interviews (including oral test results) while focusing on the listening bottleneck in context-poor FLD. 

## 2. Research Background

### 2.1. Different Context Schools and Views

Prior context models and underlying views tend to focus on parts rather than a whole. One of the views in the Socio-linguistic School is Language Socialization, holding that language learners are in a communicative setting in which social, cultural, and even political factors play a dominating role. In such settings, learners and the language they learn are supposed to be socialized with the help of parents/teachers, viz. socializing agents. Ochs [22] (p. 408) defines language socialization as “the process whereby children and other novices are socialized through language, part of such socialization being a socialization to use language meaningfully, appropriately, and effectively”. Only when learners participate in learning in such settings where social meanings and linguistic forms impact their understanding and use of the target language can they become competent interactants. With language viewed as a means as well as a central goal for human socialization, language resources, and interactive practices are of paramount importance to learners who need to know the various types of social status and social roles and to learn how to understand and express attitudes and emotions in the process of socialization. This model has been applied in exploring second language (L2)/foreign language (FL) (L2 and FL are normally distinguished in second language studies in the sense that L2 is learned by foreigners in the study abroad community, e.g., foreigners learn English as L2 in the USA. In contrast, FL is learned in communities where learners’ mother language is L1, which influences their FLL or FLD. However, some researchers tend to use L2 to refer to both in a broad sense. In this article, they are different terms. FLD is an emerging term that has been used to replace acquisition and learning that have been differentiated by Krashen [7] because acquisition and learning are sometimes unlikely to be differentiated in some cases) classroom conversational interactions between teachers and students. L2 learners are supposed to learn especially the discourse and pragmatic competence from their teachers [23,24]. Kasper [24] (pp. 519–520) argues that a ‘language socialization approach’ is capable of examining how L2 pragmatic ability is acquired and that much of pragmatic socialization is implicit whereas explicit socialization is also strongly evidenced by researchers such as [25,26] and others. Thus, L2 learning can profit from both implicit and explicit language socialization in communicative settings. This model, however, has no explicit view on learning context except that some sociolinguists [27,28] propose a list of mostly external contextual factors. Despite its insistence on implicit and explicit language socialization, no order parameters are found concerning sequenced implicit-explicit awareness.

Another socio-linguistic view is more language-based, represented by Halliday and Hasan [5]. This model views human learning as a process of making meaning—a social semiotic process; and language as the ontogenesis of learning as well as the ontogenesis of language. It is the essential condition of knowing, the process by which experience becomes knowledge rather than a domain of human knowledge. Learning can be interpreted as inherently a semiotic process in which two constraints are identified: one is that theory is based on language that is unaware, not self-monitored; in context, not in a vacuum; observed, not elicited; the other is that the theory should integrate the system with the instance: language with text, language with parole, competence with performance [29]. L2/FL learning is viewed as a process of resocialization as its learning starts with the help of Language 1 (L1) knowledge, and after learners master an amount of L2 knowledge, they start to transfer to L2 and continue to develop the meaning potential in L2; it is success rather than perfection that should be emphasized and prioritized [30].

Halliday’s context view is more explicit, starting with linguistic context, and extending it to situational and cultural contexts. Regarding the relationship between context and language as realizational, Halliday [5] (p. 49) synthesizes text, context, and learning for FLD, with a focus on five periods in the cycle of text and context: the text as a metafunctional construct, the context of situation, the context of culture, the ‘intertextual’ context, and the ‘intratextual’ context. He also emphasizes that “all learning is a process of contextualization” [5] (p. 49). Such a view helps understand how language acts on social contexts and how social contexts affect language use and language learning. 

In sum, the Socio-linguistic School focuses on the relatedness of language to sociality. However, some limitations to the Socio-linguistic School may lie in its underestimation of cognitive and psychological factors in language learning and the difficulties in its applications to FLL in ‘socializing agents’-poor settings. Halliday’s model can tell us quite a bit about ‘input’ and the ‘target language’ but much less about ‘intake’ and ‘online processing’ as pointed out by [31], ignoring learner individualities, especially learning initiative and learners’ L1 external and internal contextual knowledge.

The Cognitive School as the mainstream school in SLA, in particular, traditional cognitivists, sees the mind/brain as the locus of human thought and learning, and thought and learning of this type as an information-processing form. Cognitivism concentrates on aggregates of individuals and then construes processed results as representations of “average human being[s]” [32] (p. 97). Language comprehension and production are viewed as encoding and decoding, and language processing can be studied via decompositionality, i.e., decomposed parts, if reassembled, amount to the whole. Such tenets can be seen in some of the cognitive linguistic theories, e.g., relevance theory and its cognitive context view, in particular, Chomsky’s linguistic theory. The latter had a great influence on the cognitive school in SLA, viz. its innate view on linguistic competence regardless of performance in context. Thus, SLA tends to be viewed as a mental process [33]. Knowledge must be radically decontextualized, i.e., a form found abstractly represented in the mind/brain.

Relatedly, being influenced by the cognitive decontextualization view and cognitive psychology, cognitive linguists then view context as a psychological construct, known as Cognitive Context [34]. They see an individual’s total cognitive context as the set of all the facts that one can perceive or infer. In linguistic communication, the speaker and the listener share the cognitive context, a mutual cognitive environment. That is, the same facts and assumptions are manifest in the cognitive environments of the two. They further argue that the degree of relevance of utterances depends on contextual effect and processing effort, and that contextual effect is directly proportional to relevance, while processing ability is inversely proportional to relevance [34]. Given that they interpret the processing effort as the brainpower consumed to recognize the linguistic environment, context is considered ‘dynamic’ as opposed to traditional ‘static’ context because the generation and understanding of interlocutors’ utterances continue to yield new context in online interaction. Thus, relying solely on the traditional view of context is not sufficient to convey online information completely. However, the cognitive context as a theoretical construct is incomplete because it ignores the confinement of the social and physical environments/contexts to the understanding and production of utterances and the influence of language/texts in use on social, psychological, and even physical environments/contexts. Furthermore, it also neglects the relativity of dynamic context.

The Sociocognitive School is mostly influenced by the Sociocultural Theory which focuses on the role of interaction in cognitive and language development [24] and endeavors to synthesize cognitive and social/interactional elements. Its fundamental principle is that there are different means for human cognitions such as instruments, semiotic systems (including linguistic systems), and social interactions. Human interaction itself is mainly realized through verbal interaction. Individual development commences in social relationships through participation in social-communicative activities and proceeds from these to the psychological ones, i.e., from intermental to intramental activity. One of the most important factors affecting psychological development is mastering our human experience passed on by human languages [11]. Human activity is best understood only in its sociocultural context [35]. Language functions as a means for communication as well as a tool for thinking [22,24].

Regarding SLA, Ref. [23] pinpoints that Sociocultural Theory offers one way of viewing the language learning process as one connecting sociolinguistic with psycholinguistic contexts and outcomes. Its application to SLA enriches the Interactionist theory that has developed into two main types: one is the ‘cognitive interactionist theory’ that views acquisition as a product of the complex interaction of two equally important factors, i.e., ‘the linguistic environment and the learner’s mind’ [36] (pp. 295–297), [37] (p. 243). The other type of interactionist theory is ‘more social in orientation’ and “many cognitive interactionist theories also see social interaction as the primary mechanism of mental reorganization.” [37] (pp. 243–244). Thus, both theories emphasize the sociocognitive/sociopsychological interplay in language learning. 

The sociocognitive context view proposed by van Dijk [10] after criticizing Halliday’s register theory, treats context as a mental construct, organized by schemata. This view also persists in dynamic and stratal features of context in which a knowledge device plays a strategically managing role in the appropriate use of language. As for socio-culture and the relations between context and discourse, van Dijk explores little except complexity or presupposition of cultural variations and discursive information as input. Despite its attempt for a holistic framework of context, it is essentially a mental context without taking in linguistic and physical contextual factors. Thus, it still lacks a holistic construct of context from the perspectives of timescales and temporal ranges. Sociocognitivism/interactionalism neglects the consideration of linguistic features as well as linguistic functions and their relations to contexts. Further, predictable processes of language development and orders of acquisition are not input-related alone [38]. Sociocognitive processes should be involved with not only social and physical environments but also biological ones. Biologically, cognition, perception, and motor action are integrated activities and cognitive representations are embodied and action-oriented [39]. 

### 2.2. Application Studies of Context Views in L2 or FL Development

Given the above conflicting context views, L2 studies have come up with different understandings and inconsistent empirical findings on learning context in the field. In a broad sense, studies show that the effect of learning L2 in the target language countries is greater than that of FLL in classroom instruction at home, especially in terms of communicative strategies [16], narrative abilities and semantic density [14], and fluency [15], except lexical and grammatical development [40]. Furthermore, recent research shows that the L2 learning context has a better effect than the FLL context as far as proficiency is concerned but similar learning effects in the created international speech community in at-home settings are also achieved [17]. Even in a six-month study abroad target language country, there are significant differences in the use of the pragmatic marker, like, between the university study group and the au-pairs group, with the latter’s function typology found better, and closer to the native speaker group’s [2]. 

In a narrow sense, however, the role of external/physical context on interaction is still controversial in SLA. Unlike some researchers’ [18,19] claim, interaction may not be context-dependent and “may not vary depending on whether the participants are in the classroom or the laboratory” [1] (p. 601). On the other hand, researchers report that the age factor or early foreign language learning as a key context variable, has also witnessed incompatible empirical findings. For instance, early, mid, and late learners did not demonstrate differences in FLL after a given period of time [20] while others had opposite findings [21]. Such conflicting findings and views may handicap FLD. Context variables that do not interact with one another do not have fair context effects. To elucidate the above issues, this article presents an ecointeractive context-oriented approach/perspective and a route to healthy and sustainable FLD in what follows. 

## 3. Towards an Ecocontextualized Approach

### 3.1. A Tiered Ecointeractive Context-Oriented Perspective to FLD

**Figure 1:**
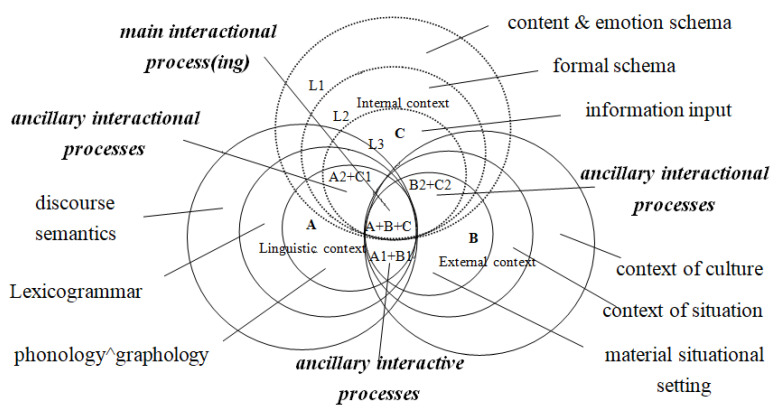
The Ecointeractive Context Model (^ = ‘followed by’).

Under the above-reviewed conditions, this article, from a healthy/sustainable development perspective, holds that FLD needs ecocontextualization as a “prescription” to treat its “disease”. Dialectically, things change through mutual interactions. The same is true of language learning. Language learning is an ecocontextualized process in which meaning is made via interstratal and intrastratal interactions . Similar to the well-known semantic triangle (concept, word, and thing), the universally accepted three categories of meanings, i.e., conceptual, textual, interpersonal/associative, cf. [5,41], may be related to their respective contexts (viz. internal, linguistic and external) as meanings emerge from context and context effects will not take place without the tripartite interactions. Additionally, meanings are expressed through symbolic means, either verbal or nonverbal. Thus, an ecointeractive context model (ECM), is composed of three types of context variables, viz. internal, linguistic, and external ones (see Figure 1). If all meanings are made verbally and/or nonverbally, then different verbal and/or nonverbal input in interactive contexts may activate and/or re/formulate different representations. Different activated representations in turn influence the construal and production of discourse.

Ecologically, language and context are symbiotic and the rationality of a learning context depends on its holistic construction through the interaction between language(s) and environments. The co-occurrence of cotextual (textual, paralinguistic, and gestural) and contextual (sociolinguistic, pragmatic, and emotive) variables facilitates meaning-making and understanding of spoken interaction [42]. The situational model [43] also believes that sharing multi-dimensional representations (i.e., space, time, causality, intentionality, and reference to main individuals) while aligned with linguistic representations in the process of communication and comprehension can be conducive to understanding. Thus, the ECM that values cotextual and contextual interaction and alignment is assumed to sustain FLD. As shown in Figure 1, the main interactional process (A + B + C) is the main processing effect of the tripartite interactive context while others (B + C, A + C, A + B) are ancillary or less interactive. In dynamic interaction, holistic relevance leads to a better comprehension of meaning via the processing and priming of contextual and cotextual variables. For instance, multimodal input excels mono-modal input in listening comprehension and vocabulary learning [44]. More importantly, the ECM is interstratal/intermental and intrastratal/intramental interaction-oriented, which will be elaborated below.

### 3.2. The Interactive Strata and Relationships

#### 3.2.1. Stratification of External Context in ECM

The external world is stratified as indicated in the hierarchical society and animal world. The same is true of the external context which can be tristratified: the physical, the situational, and the cultural. “[T]he actual physical setting in which a text might unfold” is viewed as the material setting [45] (p. 108). Such an actual physical setting contains time and space, human and nonhuman entities and their attributes, any ongoing nonverbal activities or states, and any other circumstances [46]. As [45] observes, the physical always contains elements that are not part of the context of situation. Yet there is overlapping which may vary according to the mode of discourse in the unfolding of the social process. She finds that in the physical setting where language is primarily ancillary to the social activity, the physical elements will be of high relevance and are likely to be involved in the context of situation. Otherwise, the case would be opposite [1,45,46], as indicated in the B + C processing.

The situational stratum is the one that points up to the cultural and down to the physical. Halliday [5] identified three variables for situational context, i.e., field (what is happening), tenor (social roles and role relations), and mode (what part the language is playing) that correspond, respectively, to the three metafunctions/meanings, i.e., ideational, interpersonal, and textual. Although the three contextual variables can be essential, and the linguistic realization of any of these factors depends on its combination with the other two [47], this context view has a closed rather than open list of contextual variables, excluding some other important ones as proposed by [27] (Hymes [27] identified eight components: setting, participants, ends, act sequence, key, instrumentality, norms of interaction and interpretation, and genre that form the acronym “SPEAKING”) and [48] (Lyons [48] then proposes six contextual variables which include role and status of the participants, time and space, formality, instrumentality, topic and attitudes of the participants towards it, and scope of topic). For instance, the variable, ‘formality’, which should be included in the situational stratum pinpoints the necessity for a situational stratum as formal language aligns with formal context, and informal language, informal context. Informal language is not interchangeable with formal language in use.

The cultural stratum is a broader background against which a text is interpreted. Halliday [5] relates it to the context of situation in terms of potentiality, ranging from the cultural potential to instantial situations with situation types as intermediate constructs. A situation is an instance of culture. In the ECM, it can be viewed as something out there in the speech community, a part of the collectively-shared cognition, abstract and socially-constructed. The context of culture can be realized non/verbally. Nonverbal realization can be physical/visual ‘things’ at the lower stratum, e.g., statures, pictures, clothes, and more, while the verbal one is carried/instantiated by culturally-loaded words/texts. From the perspective of learning, it is not only an interorganic process but an intraorganic one as well in which a cumulative cognitive schema is built up through the tripartite ecointeractions. The lower the strata are; the more concrete they are, or vice versa. Thus, FLD may best begin at a young age and at the lower strata (the phonic and the physical) based on the interactive context model [49], which will be elaborated later.

#### 3.2.2. Stratification of Linguistic Context in ECM

The view that language has content and expression planes exists in prior linguistic studies [47,50,51]. The content plane concerns the construal of meaning, while the expression plane, the non/verbal realization of meaning [51]. The content plane is further stratified into lexicogrammar, and discourse semantics. Given this, language is a tristratal system, viz. discourse semantics, lexicogrammar, and phonology/graphology [47]. They correspond to meaning, wording, and sounding/writing, respectively. Text is seen as the basic unit realized in sentences. A text is an instance of social meaning in a particular situational context (ibid). Language is a resource organized into three strata differentiated according to the order of abstraction. The three strata are related through realization. Discourse semantics is realized by lexicogrammar, that is, grammatical structures and lexical items; and lexicogrammar is realized by phonology or graphology [52]. Within the strata of language, the higher stratum provides an environment/context for the lower stratum.

Unlike the systemicians’ onion-stratified language-context relation, cf. [51], the linguistic context variables in ECM eco-interact and align with the other two types along a multidimensional continuum of variables between the internal and the external. The three of them circulate via ecointeraction and sustain languages and lives from generation to generation. They are interconnected at each stratum. Language helps an individual’s internal cognition align with social/physical ‘things’ from lower to higher strata, which is essential for young FLD beginners to map sound (i.e., phonic expression, the physical foundation of language) with meaning first via object-sound linking. Evidence for this has been found in some empirical studies, e.g., words with high ‘body-object interaction’ ratings are recognized faster than those without [53]. A young beginner’s implicit sound-meaning mapping prioritized FLD turns out to be more successful than the ones’ explicit form-meaning mapping prioritized FLD (beginning with reading, speaking, listening, and writing simultaneously) in a decade-long case study [49]. It is wondered whether longitudinal and quantitative studies will gain the same result when interacting and aligning with tripartite stratal representations from low interstrata (low cognitive levels of younger learners, concrete objects, implicit sound-meaning mapping) to high interstrata (high cognitive levels of older learners, abstract contents, explicit formal learning). If so, FLD will gain a healthy and sustainable route.

#### 3.2.3. Stratification of Internal Context in ECM

The internal context also has three strata: information input, formal schema, and content schema. The latter two are cognitive schema [54]. The lower strata activate the upper. Kern [55] further exemplifies that formal schema is concerned with the knowledge of applying language, while content schema, subject knowledge about the reality in the world, and cultural concepts. The two schemas include subschemas, ranging from audio, video, and structural schemas to conceptual, individual, environmental, procedural, and emotional schemas.

The cognitive context model views context as a mental construct concerning the listener’s subsets of assumptions about the world [34]. The study of cognitive context is deprived of considerations of the physical context which plays an important part in the dynamic interaction among contextual and cotextual variables. This point is supported by the change of the concept of discourse domain from a personally and internally created cognitive construct [56] to “a cognitive construct created in response to several factors, including semantic category, but also to other features of situational and linguistic context” [56] (p. 27). Such a change is influenced by both Fishman’s [57] sociolinguistic domain and Zuengler’s [58] interactionally-negotiated view. Furthermore, due to the different interactional ranges, three linguistic domains with different sizes (L1, L2, and L3) are marked by dotted lines in Figure 1. One point to note is that one’s L1 and L2/FL schemas, though complementary, are proven to have different incidences on FL production [59].

#### 3.2.4. The Intrastratal and Interstratal Relationships

**Figure 2:**
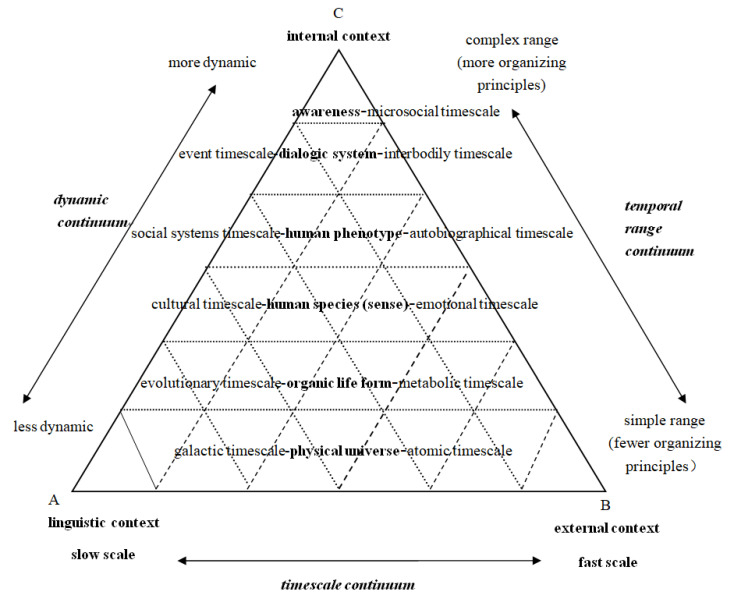
Tiered triangular network in the main interactional process(ing).

The relationship between strata within each context circle is intrastratal. Such intrastrata consist of different features of language(s) or signs with different representations. They are the intra-systems of self-organizations that interact with one another in a circulating triangle (cf. Figure 1). Thus, they have different strata, timescales, and temporal ranges respectively, some of which are immediate and relevant (e.g., awareness, dialogue), and others are distant and less relevant (e.g., organic life form and physical universe) in the main interaction process(ing) (cf. Figure 2).

The internal context connects both external and linguistic contexts interactively. The interstratal relationship among the three contextual circles is interdependent and ecologically symbiotic. Mental representations link and ground themselves on the outside world. The latter, including the collectively formed ideological and natural world, shapes an individual’s mental representations. Language can be viewed as an expression of meaning as well as a reflection of the external world. In this sense, well-educated adult individuals’ mental representations are shared reflections of collectively formed linguistic, cultural/ideological, and natural knowledge. The relationship between the internal context and the external context is reciprocal, and so is the one between the internal context and the linguistic context. Thus, the relationship between context and language is realizational and symbiotic, and the linguistic system (language) can be instantiated by text (languaging).

#### 3.2.5. The Main Interactional Process(ing)

Interaction arises between at least two types of context variables (cf. the ancillary interactional processes in Figure 1). The main interactional process(ing), however, requires three, forming an interactive context. Figure 2 provides more clearly the tripartite contextual variables across temporal ranges and timescales that are drawn on [60]. In the ECM, the main interactional process(ing) can be a specific tiered triangular network in which the internal context is located at the top, activated by the physical and/or linguistic variables from the bottom of the simple temporal ranges. The entire tripartite relevant context variables are interrelated more or less to the small triangles in the big triangle, i.e., the interactional process(ing), as only relevant temporal ranges and timescales may be involved. In the main interactional processes, there are three continuums, i.e., the timescale continuum, the temporal range continuum, and the dynamic continuum. Those on the left of the ranges (from ‘awareness’ to ‘physical universe’) are slow whereas the ones on the right are fast. The complex range near the internal context has more organizing principles while the simple range near the external context has fewer. The same is true of the dynamic continuum which elucidates the traditional conflicting views between the ‘dynamic’ and ‘static’ contexts in the literature.

Each triangular interaction of the temporal ranges and timescales is governed by awareness, emotions, and more in the internal context. Awareness interacts dynamically with the other two types of related variables to form a communicative context. Simultaneously, although influenced by external variables, awareness has an active role, which is indicated by the inverted triangles in Figure 2. The triangle and its role, big or small, not only occur in the communicative context (main interactional processing), but also extend to auxiliary interaction, linguistic context, and external context (Figure 1 and Figure 2). Content/emotion schema comes from the complex awareness side of the internal context. The more cognition intervenes, the more internal contextual parameters are involved in the interaction or meaning-making. In the external context, collectively shared and socially distributed cognition stored in the brains of the human species can be transformed into extended cognition, i.e., external/physical environments viewed as part of cognition under the condition that the latter are processed in the brain [61]. The fundamental aim of cognition is to adapt to the environment [62] that is structured in terms of cognitive activities. Similarly, as for linguistic context, cognition is language-based, forming a meaningful semiotic map [52], which is essentially extended cognition, in that meaning derives more from the linguistic context, and a language with slow timescales evolves into different ‘forms’ and carries meanings of multidimensional simultaneity, both historical and sociocultural.

The dynamic relativity of the ECM is prominent and consistent with the recent research on extended cognition and embodied cognition that differs from traditional cognition. Context is relatively dynamic because it is realized by phonic or graphic output which in turn becomes part of the external environment as representations/input together along with ancillary gestures and paralinguistic symbols (embodied cognition). In spoken interaction, rapid relevance of phonic utterances to meaning via internal processing can be more dynamic (as with object-meaning processing), whereas slow relevance in the interaction with/of written texts with sociocultural meaning at the slow timescales, can be less dynamic. Let alone the evolution of organic life forms and the interaction (e.g., human languages’ broadcast) with other humans or creatures in outer space at galactic timescales of the physical universe. In sum, characterized by the dynamic continuum (Figure 2), the composing of context is relatively dynamic, interacting with the three types of contextual parameters which are mutually preconditioned, co-relative, and indispensable.

### 3.3. A Route to Sustainable FLD: Sequenced Implicit/Phonic-Explicit/Graphic Processing

The ECM presents a clearer picture of how L1 and L2/FL contextual variables interact with one another in their interactional processes. In the process of contextualization, L1 and L2/FLL learning contexts differ in the way they are situated in social interactions and based on what functions they have in their respective contexts. L1 is more socially contextualized and has more social functions than L2/FL. FL learners learn FL in their L1 and culture-dominated external environment. As a result, they learn FL mostly through interacting with intuitively written target texts in textbooks (i.e., linguistic context) rather than with real native speakers or their authentic texts in immediate contexts. Many learners still have listening comprehension bottleneck even after learning FL for over a decade. Under such circumstances, how to work out a route to healthy/sustainable FLD is a major objective herein.

**Figure 3:**
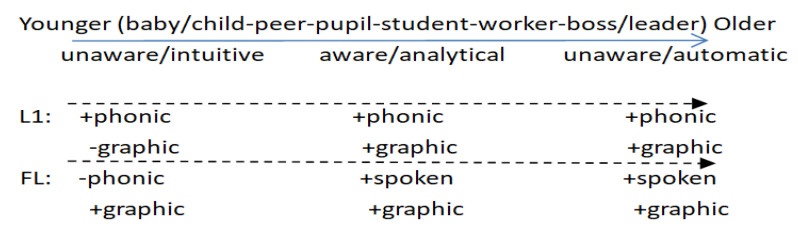
Different L1 and FL contextualized processes.

Based on the ECM, the tripartite contextual interaction processing enables forms and functions to be mapped via the abstraction of symbolic units from examples in the FL context. Such mapping aligns interstratally and intrastratally (including intermentally and intramentally). Interstratal interaction means the incorporation of internal, linguistic, and external contextual variables and intrastratal interaction means the ascending order of language learning from lower strata to higher strata, ranging from implicit/phonic/physical/early stage to explicit/graphic/abstract/late stage. In this developmental process, the phonic expression of meaning is mainly implicit whereas the graphic expression of meaning is chiefly explicit. Such differences can be found between L1 and FL learning. Regarding explicit and implicit learning, L1 learning can be classified into three stages in its socializing process: (1) the unawareness (phonic) stage of learning language, (2) the awareness (graphic) stage of learning about language, and (3) the unawareness (automatic) stage of learning through language (see Figure 3).

At the first unawareness stage, children are exposed to the phonic input in L1 without any conscious learning of the language form. At this phonic stage, they try to understand meanings by constructing links of sounds with meanings (concepts, signs/things) through verbal/nonverbal interpersonal interaction with adults/parents. They are being illiterate or ‘cognitively underdeveloped’ primeval roles (baby/child/peer) as they cannot read or write, which is the sound-meaning mapping or the intuitively contextualized physical-phonic stage. At the second stage, learners gradually notice and generalize “rules” such as plural nouns, with the help of increasing awareness as they become more socially experienced and cognitively developed. They start to learn how to read and write, which is subject to the interaction with written language/genre in a linguistic context, though the interpersonal phonic interaction still continues. Learners’ conscious/analytical learning of the written language dominates at this stage. They learn how to spell words, make sentences, and compose articles of different genres from sample texts which mostly take place with the assistance of teachers at school. Noticing facilitates language learning [8,63]. (cf. without written texts in linguistic contexts, learners cannot learn written language/genre directly from teachers, which explains why linguistic context is essential in the ECM). At the third stage, after mastering the grammar system, together with its social functions, learners become gradually unaware of the language, which is the automatic stage. They learn other subjects such as geography, physics, and more through language. Notably, unawareness takes effect all the time in L1 spoken interactions despite adults’ occasional corrections during early stages. 

In contrast to L1 learning, most FL learners begin with the awareness stage, even at an earlier age. They start directly to learn to read and write more consciously at the very beginning rather than to set up links of sounds with meanings unconsciously. As noted previously, phonic expressions and physical objects at the lower strata (the expression plane) that are more concrete are prone to learning, needing less cognition. Thus, the unawareness of FLL is removed from the early stage in the FLL context. This is probably one of the decisive factors for the unsuccessful FLD of most learners. A functional-semantic interpretation for this is that nonverbal meaning-exchanging and functions take place much earlier than verbal ones. Similarly, the meaning-exchanging and functions in phonic L1 take place much earlier than the ones in graphic L1. That Hasan [5] distinguishes phonic from spoken is significant and instructive in the sense that in FLL the spoken language can be still ‘written’ or graphic rather than phonic. Thus, at the second and third stages, phonic is replaced by spoken in Figure 3 in that graphic spoken language is learned simultaneously with reading and writing in the FLL context. Such FLL has led to explicit or rote learning of the language, which has been ignored so far by educators, textbook designers, and teachers. In this sense, unconsciously learned spoken languages are supposed to be interactional and phonic rather than graphic, which belongs to the lower strata of the ECM aligned with implicit awareness, concrete objects, and low roles in immediate contexts. In sum, the above analysis pinpoints that a route for sustainable FLD consists of the three orderly stages (i.e. order parameters): phonic (unconscious learning FL) stage, graphic (formal/conscious learning about FL) stage, and automatic (unconscious learning though FL) stage, which is aligned with learners’ roles and cognition from low to high or concrete to abstract.

In compliance with the above objectives and contentions, three hypotheses are made below to testify whether there is a sound-meaning mapping prioritizing route to sustainable FLD from the ecocontextualized perspective, i.e., the learning trajectory from physical-phonic stratum (unconscious learning FL) up to abstract-formal/graphic (conscious learning about FL) and the automatic (unconscious learning through FL) content-based stratum.

**Hypothesis** **1.** *There is no homogeneity between NSMMP and SMMP learners of both senior high schools and universities in terms of their oral and written English proficiencies at the early stage.*

**Hypothesis** **2.** *There are no significant differences between the NSMMP and SMMP learners at both senior and tertiary levels in terms of oral and written English proficiencies.*

**Hypothesis** **3.** *If the above two hypotheses are true, there are no significant differences between NSMMP and SMMP learners in their scores in entrance examinations, interview/oral tests, and multiple choices.*

## 4. Materials and Methods

### 4.1. Study Design and Recruitments

Considering the hypotheses and the time span of the order parameters discussed above, this study adopted a cross-sectional design and a structured questionnaire for surveys, followed by an interview as immediate experiments or longitudinal and quantitative studies are unlikely to be carried out in different schools or universities over a decade. Furthermore, based on the ecocontextualized view and the SMMP route to FLD, the study design implemented a cross-sectional comparison among juniors, seniors, and tertiary participants via questionnaire and interview/oral test due to the potentially limited population of SMMP learners. It is hoped, thus, that in so doing, learners’ different age, cognitive levels, and contextual knowledge of lower/higher intra/interstrata will be highlighted and thus their effects on SMMP vs. NSMMP FLD will be displayed more clearly.

The questionnaire collection sites were intact parallel classes in Guangzhou city, China. 281 students in total were recruited on a voluntary basis after being given a full explanation regarding the purpose of the study by the research assistants of the project. Among them, there were 99 middle school students (69 Senior 2 school participants for a paper questionnaire and 30 Junior 2 school participants for the interview/oral test). 182 university students filled out the questionnaire forms online via WeChat. While most FL learners began with letters, i.e., reading, speaking, listening, and writing simultaneously, students from an experimental high school in Guangzhou, one of the national key middle schools, could be an exception, which helped with a potential collection of the data required herein. According to the students’ answers to demographic questions, they were divided into NSMMP and SMMP early starters. The inclusion criteria were as follows: All the students were able to communicate in Mandarin and complete the questionnaire written in Chinese. The exclusion criterion, as explained by the research assistants to the participants, was that students who moved from English-speaking countries to Guangzhou with their parents were excluded due to their L2 or L1 acquisition environment during childhood.

### 4.2. Questionnaire and Interview/Oral Test

The study’s questionnaire consists of 6 sections. Section 1 (items 0–4) is basic demographic information, including sex, age, duration of exposure to spoken English, relationship status, and current residence. Section 2 (items 5–9) is about early-stage learning performances including oral proficiency (i.e., listening and speaking aiming at sound-meaning mapping) and fundamental written processing abilities (viz. spelling and reading reaction). Section 3 (items 10–16) is about oral language proficiencies, including listening and speaking. Section 4 (items 17–23) focuses on written language proficiencies, i.e., reading and writing abilities. Section 5 (items 24–25) concerns the senior high school and university entrance examination scores. Section 6 (items 26–38) is multiple choices testing learners’ language proficiency, partially derived from sample sentences from [64,65] and the language-sense questionnaire by [66]. The Likert 5-point scale was adopted, using a scale ranging from 5 points to 1 point. A pre-questionnaire was carried out for the validity of the items, and consequently, two unreliable items were deleted, with 38 items left in total. The total oral and written scores were obtained through summation of listening and speaking (oral) scores and reading and writing (written) scores respectively, with higher scores representing higher levels of oral and written language proficiencies. The Cronbach’s alphas of the total scores for early and late oral and written proficiencies in this study were 0.83 and 0.87, respectively.

The interview was carried out in a foreign language school. 30 juniors were recruited from a grade, among whom fifteen were randomly selected from those who reported that they were SMMP early starters whilst the other fifteen, NSMMP early starters. Before the interview, they were kept in a separate room. Each was separately called into the interview room and was asked in turn the same four questions. To examine how fluently and correctly the students answered, two testers, while recording response-reaction time, assessed the testees’ responses and gave marks according to the evaluation criteria. Average marks of the two testers were calculated for the participants whose SMMP or NSMMP types were unknown during the test. The interview was designed to compensate for the self-reported questionnaire items.

### 4.3. Statistical Analysis

This study adopted the SPSS for data analysis. First, descriptive statistics were adopted to display basic demographic information. Next, Independent Sample Analysis was used to explore the mean values and t values for the early and late oral and written proficiencies of the four cohorts. These values were calculated for listening and speaking (oral proficiency) as well as reading and writing (written proficiency). All the entrance examination scores the participants filled in the forms were converted into percentages as some examinations have different total scores. For some reason, only 100 students filled the questionnaire examination items correctly (others failed to fill their senior high school entrance examination scores or university entrance written/oral examination scores). Finally, the *p* values of the proficiencies at early and late stages were calculated so as to analyze potential significant differences between the NSMMP and the SMMP learners of both secondary school and universities.

## 5. Results

### 5.1. Early Stage Survey Results

Table 1 shows there were no significant differences between senior school NSMMP and SMMP learners in the oral proficiency, *p* (t) = 0.34 (−1.06) and written proficiency, *p* (t) = 0.48 (−0.77). There were no significant differences between university NSMMP and SMMP learners in either the oral proficiency, *p* (t) = −0.71 (0.66), or written proficiency, *p* (t) = 0.77 (−0.33), indicating that all the participants of different levels agreed that they had homogeneity in either oral or written FLL at the early stage though they had different time spans and routes of FLL.

### 5.2. Late Stage Survey Results

Table 2 indicates significant differences between senior school NSMMP and SMMP learners in all the oral proficiency, *p* (t) = 0.00 (−5.11), and written proficiency, *p* (t) = 0.00 (−5.77). Similar gains were also found between university NSMMP and SMMP learners in both the oral proficiency, *p* (t) = 0.00 (−5.49); and written proficiency, *p* (t) = 0.00 (−4.69). These results may indicate that they can be subjective based on the questionnaire items and thus more convincing results need to be found from other data collections such as tests in what follows.

### 5.3. Interview/Oral Test Results, Elicited Language Abilities, and Examination Scores

Table 3 shows that there were significant differences between junior SMMP learners and junior NSMMP learners in oral fluency, *p* (t) = 0.009 (−2.22), whereas there were no significant differences in their oral accuracy, *p* (t) = 0.418 (−0.82). As for the tertiary learners’ multiple choices of translation and grammar, no significant differences were found between the two types, *p* (t) = 0.063 (1.89); *p* (t) = 0.97 (1.68) though the NSMMP early starters were better than the SMMP ones. These results corroborate the SMMP route to FLD, i.e., SMMP learning can be more sustainable than NSMMP learning as sound-meaning can be well mapped by early starters if they solely begin with sound-meaning mapping rather than simultaneous reading, speaking, listening, and writing.

Table 4 shows that significant differences were found between NSMMP and SMMP learners in Senior High School Entrance Examination scores, *p* (t) = 0.034 (−2.167), and University Entrance Oral Examination scores, *p* (t) = 0.032 (−2.187), except University Entrance Written Examination scores, *p* (t) = 0.577 (−0.561). These results indicate that NSMMP learners turn out to have the same learning achievements in written language as SMMP learners whereas they cannot achieve so high a proficiency as the latter in terms of oral English. Another indication is that scores can be more reliable than elicited intuitive judgments as shown in the tertiary written results from questionnaires (Table 2).

## 6. Discussion

Addressing Hypothesis 1, our finding reveals that there were no significant differences between the NSMMP and SMMP learners of both senior high schools and universities in terms of the oral and written English proficiency survey at the early stage. Thus, Hypothesis 1 was rejected. Although the learners had different lengths of FLL, the two types of learners of either senior high school or universities were at the same level from the outset. Such a design validated the results. One more point is that all the SMMP learners reported that they had at least six months of SMMP learning before beginning with FL reading and writing. Of all, some learners started learning English as FL at Primary 3, especially those from the countryside; some started at Primary 1 in cities; some started before primary school, most of whom were SMMP learners.

No matter what types of starters the learners are, their interaction between internal and external/linguistic context in FLD is limited because it is not authentic but organized and confined to the classroom settings. Moreover, due to the lack of native speaker teachers, FL teachers who are mostly not proficient enough or native-like, rely heavily on teaching materials, textbooks, audio-video tapes, and more in the physical context, which helps avoid their weaknesses (lacking proficiency). Generally, language learning through interaction in rich interactive contexts facilitates implicit learning of implicit knowledge as seen in context-rich au pair or international community FLL settings [2,17]. Conversely, FLD in context-poor settings as in FLL classrooms may need some explicit learning of implicit knowledge [67], especially for adult learners. The encoding of implicit knowledge such as spoken language features needs shared contexts (e.g., shared cognition and community activities). Such shared contexts include early starters’ implicit awareness and intuitive imitation, which requires teachers to share the implicit spoken language through implicit SMMP interaction with them rather than to teach explicitly through NSMMP interaction. As young starters are cognitively underdeveloped, the proximate context—the learning-sustaining configuration of learners’ age, roles, low cognitive levels, and implicit awareness or imitation in alignment with phonic interaction is assumed to play a part in FLD. FL Teachers, though native-unlike, can expose young learners to story-telling videos or cartoons while asking questions or helping with listening difficulties in that such measures help early starters map sound and meaning. In doing so, raising context-language awareness (implicit matching videos or physical objects with sounds) simply follows the SMMP route to FLD as it may be the training of an intuitive ‘native ear’. Hence, given the slow SMMP learning process, the result that no significant differences between the SMMP and NSMMP learners were found at the early stage may be chiefly due to the short learning period of time, and a longer period is needed to examine the learning outcomes.

Addressing Hypothesis 2, our finding indicates significant differences between NSMMP and SMMP learners of either the senior high school or the universities in terms of both oral and written English proficiencies. In contrast to Baumert et al.’s [20] findings that there were no differences among early (Year 1), mid (Year 3), and late (Year 5) starters after a few years, our finding is more significant for its basis on the learning mechanism, i.e., SMMP vs. NSMMP while theirs was lack of differentiating methods. This mechanism is more conducive to FLD as it caters to younger learners’ configurations of interactive context variables, which means that the related (biological, physical, linguistic, cognitive, mental, and social) variables coordinate sequentially in interaction. Evidence first comes from genetic/neuro research findings: As reported in the journal, Nature, one of the 2500 units of DNA that make up the FOXP2 gene had a mutation that prevented it from forming the normal genetic order needed for early brain development, leading to language problems [68]. It was also reported that different brain areas had different linguistic functions, namely, the left frontal lobe is responsible for the cognitive memory of words while the right frontal lobe is responsible for the cognitive memory of images. Patients whose left hemisphere’s projection cortex, the Exner area, is damaged are found unable to coordinate head, eye movement, and hand movement, leading to agraphia [69].

Cognitively, young starters, due to their low cognitive level, are unlikely to process complex information with the four skills simultaneously involved in learning. If forced, they will be unable to comprehend and produce the target language automatically at the third stage. Interacting with the intuitively produced graphic/written language during the onset stage belongs to the less dynamic end of the dynamic context continuum rather than the more dynamic phonic one, viz. sound-meaning mapping, which makes a huge difference in processing. Thus, this finding may have provided empirical evidence for the acquisition hypothesis [7] that has been considered difficult to be verified.

Given the FLD route, a good beginning presupposes an early age and prioritizes the phonic learning stage. One possible reason is that the material/physical objects are always situated in the early starters’ learning, particularly the phonic elements, i.e., the material base of language. One point to note is: if the SMMP early starters had more gains in oral proficiency, why did the results also show more gains in written proficiency than the NSMMP early starters? Generally, written language learning requires more analytical abilities at which the SMMP starters are assumed not to excel. Our interview may prove it. The junior SMMP students had two advantages that the NSMMP junior students did not possess. One is that their fluency was better, and the other is their reaction time was quicker in conversations, indicating that they had better oral proficiencies. However, their accuracy was not as good as their counterparts’. This finding is even hidden in their score on Senior High School Entrance Examination (cf. Table 4). Thus, this question necessitates further direct-data-based discussion below.

Addressing Hypothesis 3, our result reveals significant differences between the NSMMP learners and the SMMP learners in their oral English scores (also fluency test results) rather than written language scores for the juniors’ interview (accuracy test results), tertiary entrance written examinations, and translation and grammar (discrete) multiple choices. However, despite their strength in oral proficiency, the SMMP learners could learn well the written language, especially grammatical knowledge explicitly as the NSMMP learners did at the late stage, as there were no intergroup significant differences in the written language results. This finding indicates that listening and speaking can be better learned implicitly without reading and writing involved at the initial stage. Hence, such an SMMP route to FLD has been proven more sustainable at the late stage. The results herein provide evidence for the route to FLD and support the over ten-year case study finding [49]—an FL learner who started learning FL by prioritizing sound-meaning mapping turned out to be more successful/sustainable than those who started FLL at exactly the same early age through simultaneous reading-speaking-listening-writing learning. Thus, it is not purely the earlier, the better, but well begun is half done.

Returning to the above irresponsive question, the SMMP starters’ written English proficiencies were significantly different from the NSSMP starters’ based on the self-reported questionnaire data in Table 2. However, the scores for the tertiary entrance written examination, the juniors’ interview accuracy, and the multiple choices were not so in Table 3 and Table 4. Such self-reported results may indicate (1) the subjectivity of the scaled questionnaire, and (2) the SMMP learners’ better self-efficacy than the NSMMP learners’ as most of the questionnaire items were based on five-scale-point choices, which may not be as accurate as examination marks or translation and grammar multiple choices though the latter were also juxtaposed in the questionnaire. Yet, self-efficacy can be a learning-promoting factor through peer learning, the successful learning experience of which equips students with more positive self-efficacy [70]. In this study, SMMP learners’ oral performances sounded eminent in class when answering teachers’ questions or speaking in group work, which may, in turn, enhance their self-efficacy.

Socially, young learners map sound and meaning in immediate contexts in which simple utterances, physical objects, simple social roles, and more are aligned implicitly with one another via their socializing experiences. Thus, the older they are, the more sociolinguistically appropriate their language(s) will be. Given this, language teaching should take into consideration the three stages for the FLD route in which the order parameters change. Learners of different ages and cognitive levels are bound to have different roles and surroundings to align with at the three stages. On the one hand, FLD contexts are short of native speakers of the target language, especially their social roles in society. Thus, pseudo roles (‘Pseudo roles’ refer to the social roles that learners play in learning activities as they are not real or taking place in real social communications but role-played by the students in classroom settings. Such roles as boss or worker use language in different ways. One example will suffice: A boss may deploy the discourse marker ‘so’ or the high modality words, ‘must, should’, and the like, more frequently than a worker in their conversation.) and their speech extended cognitively in linguistic contexts are essential in the FLL context. On the other, social roles and role relations are enacted by the target language to which one is exposed. The roles one plays range from baby, child, peer, roommate, classmate to worker or boss and more, which demonstrates a bottom-up trajectory of development from the low strata of contexts (implicit awareness, low-level cognition and roles, phonic expression, and physical objects) to the upper ones (explicit awareness, high-level cognition and roles, graphic expression, and abstract concept or content), a continuum in fact. Such roles are closely realized by their corresponding language at the different stages. Furthermore, social roles embody their thoughts, emotions, values (cognitive schema), and responsibilities [71]. Thus, those who do not have correct role awareness or an understanding of their own and others’ roles or their thoughts, feelings, and values may have difficulties in social communications [72]. In this sense, context-language awareness can be better raised when role-playing and aligning with social roles and their speech, which is in line with the finding that implicit learning effects depend on role relationships in the FLL context [73]. Undoubtedly, explicit learning is necessary in some contexts [74], in particular, when learners grow older with their analytical abilities developed, cf. [75].

## 7. Conclusions

This article has presented an ecocontextualized approach/perspective by integrating the concepts of existing context studies from the perspective of healthy/sustainable FLD to clarify the controversial issues on the role of context and inconsistent underlying learning theories in L2 studies. To understand the differences in the interactional L1 and L2/FL processes, this perspective has been further analyzed and discussed in terms of sequential inter-intra-stratified contextualized learning and a sound-meaning mapping prioritized route for sustainable FLD. Empirical research was conducted to verify the perspective and the route. Findings show that SMMP learners excelled over the NSMMP learners, especially in oral proficiency, and that oral, especially listening abilities, could not be developed well at the late stage by NSMMP learners, whereas written proficiency could be developed later by both. Thus, following the sound-meaning mapping prioritizing route will lead to healthy/sustainable FLD.

Implications of this perspective and route are significant as traditional approaches and teaching methods could not solve the listening bottleneck problem of most FLL students. The application of this route will promote the FLD of worldwide learners and their international communication. Thus, it is envisaged that more SMMP early starters in future research will help further corroborate the findings of the present study, and overcome the shortcoming of the limited number of participants in this study due to the restricted availability of SMMP learners at present. Furthermore, further research can be conducted on the effects of SMMP FLL early starters from different L1 backgrounds. Considering the limitations in the research methods, more sound methods such as triangulation methods can also be utilized to verify the ecocontextualized approach and the SMMP route for FLD.

## Figures and Tables

**Table 1:** Results of learners’ early stage language abilities.

Types of Learners	Listening & Speaking			Reading & Writing		
	m	t	*p*	m	t	*p*
senior NSMMP-SMMP	3.08/2.60	−1.06	0.34	3.76/3.54	−0.77	0.48
tertiary NSMMP-SMMP	2.68/2.78	0.71	0.66	3.82/3.81	−0.33	0.77

Notes: *a* = 0.05; senior NSMMP (*n* = 32); senior SMMP (*n* = 37): tertiary NSMMP (*n* = 100): tertiary SMMP (*n* = 82).

**Table 2:** Results of learners’ late-stage language abilities.

Types of Learners	Listening & Speaking			Reading & Writing		
	m	t	*p*	m	t	*p*
Senior NSMMP-SMMP	11.91/9.28	−5.11	0.00	13.39/10.42	−5.77	0.00
Tertiary NSMMP-SMMP	11.93/9.61	−5.49	0.00	12.85/10.59	−4.69	0.00

Note: *a* = 0.05; tertiary NSMMP (*n* = 100): tertiary SMMP (*n* = 82).

**Table 3:** Results of juniors’ oral test and tertiary learners’ multiple choices.

Types of Learners	Fluency				Accuracy			
	m	sd	t	*p*	m	sd	t	*p*
junior NSMMP-SMMP	80.43/86.06	4.60/6.21	−2.22	0.009	79.33/81.16	6.52/5.65	−0.82	0.418
	(translation multiple choices)				(grammar multiple choices)			
tertiary NSMMP-SMMP	2.17/1.70	1.11/1.14	1.89	0.063	4.41/3.90	1.44/1.37	1.68	0.097

Notes: *a* = 0.05; junior: NSMMP (*n* = 15), SMMP (*n* = 15); tertiary: NSMMP (*n* = 70), SMMP (*n* = 30).

**Table 4:** Entrance examination results between NSMMP (*n* = 70) and SMMP (*n* = 30) learners.

						95% Confidence Interval for Difference	
	t	df	*p*	MD	SE	Lower Bound	Upper Bound
SHSEE	−2.167	67.165	0.034	−2.992	1.381	−5.749	−0.236
UEWE	−0.561	63.196	0.577	−0.820	1.462	−3.742	2.103
UEOE	−2.187	85.258	0.032	−3.888	1.778	−7.423	−0.353

Notes: *a* = 0.05; SHSEE = Senior High School Entrance Examination; UEWE = University Entrance Written Examination; UEOE = University Entrance Oral Examination.

## Data Availability

Not applicable.

## Supplementary Materials

The following supporting information can be downloaded at: https://www.mdpi.com/article/10.3390/ijerph191610342/s1, Supplemental Material S1: Questionnaire form; Supplemental Material S2: Interview/oral test questions.
