# Supplementary material for: How Does a Healthy Interactive Environment Sustain Foreign Language Development? An Ecocontextualized Approach
Source: Int J Environ Res Public Health. 2022 Aug 19;19(16):10342. doi: 10.3390/ijerph191610342 (PMC9408107; doi:10.3390/ijerph191610342)
Supplement: Supplementary file 1 [file ijerph-19-10342-s001.zip › S1.pdf]

Supplemental material S**1**

Questionnaire form

Dear participants:

Thank you for answering the following questions in this questionnaire carefully. We assure you that your answers will be kept confidential! Again thank you very much for your support and cooperation!

0. Gender: School: Grade:

1. Have you ever attended an English kindergarten?

A. Yes B. No

2. Have you been exposed frequently to English listening and speaking like attending an English kindergarten, listening to English stories, watching English cartoon, communicating with others in English?

A. Yes B. No

3. How long have you been exposed to English listening and speaking before starting formal school English learning?

A. More than 3 years B. 2 to 3 years C. 1 to 2 years D. half to 1 year E. 0

4. When did you start formal school English learning? (including English listening, speaking, reading and writing)

A. Before primary school B. 1/2 grade in primary school C. 3/4 grade in primary school D. 5/6 grade in primary school E. Middle school

5. What about your oral English when you just started learning English? (like asking for direction)

A. Fluent without problems B. Fluent most of the time C. With a few problems D. Not

that fluent with many problems E. Not fluent

6. What about your English listening skill when you just started learning English?
A. I can react immediately B. I react quickly unless encountering something unfamiliar C. with a few problems D. I react not that quickly E. I react slowly

7. What about the accuracy rate of your word spelling when you just started learning English?
A. 80%~100% B. 60%~80% C. 50%~60% D. 30%~50% E. less than 30%

8. What about your English reading skill when you just started learning English?
A. can react immediately B. react quickly unless encounter something unfamiliar C. with a few problems D. react not that quickly E. react slowly

9. What about the ranking of your English grade in your class when you graduated from primary school?
A. first five B. five to ten C. ten to twenty D. twenty to thirty E. after thirty

10. When you speak English, will you organize English firstly in your mind or unconsciously speak Chinese?
A. never B. almost never C. seldom D. occasionally E. often

11. When you speak English extemporaneously or answer questions in English, what about your fluency?
A. Fluent without problems B. Fluent most of the time C. With a few problems D. Not that fluent with many problems E. Not fluent

12. When you listen to English records, will you firstly think about the Chinese meanings of those English words in your mind?
A. never B. almost never C. seldom D. occasionally E. often

13. When you listen to English recordings, do you comprehend lexical meanings word by word?
A. never B. almost never C. seldom D. occasionally E. often

14. What about your reaction to English recordings or listening materials? Take the listening part in Senior High School Entrance Examination or College Entrance Examination as an example.
A. can react immediately B. react quickly unless encounter something unfamiliar C. with a few problems D. react not that quickly E. react slowly

15. Do you often watch English movies?
A. often B. occasionally C. seldom D. almost never E. never

16. How much can you understand when you watch a movie without subtitles?
A. 80%~100% B. 60%~80% C. 50%~60% D. 30%~50% E. less than 30%

17. When you read English materials, do you firstly think about the Chinese meanings of those English words in your mind?
A. never B. almost never C. seldom D. occasionally E. often

18. When you read English materials, will you read out the words in your mind?
A. never B. almost never C. seldom D. occasionally E. often
19. What about your reaction to English reading materials? Take the reading part in Senior High School Entrance Examination or College Entrance Examination as an example.
A. I can react immediately B. I react quickly unless encountering something unfamiliar C. with a few problems D. I react not that quickly E. I react slowly

20. Do you often read English literature, newspaper, etc.?
A. often B. occasionally C. seldom D. almost never E. never

21. When reading English materials, how many words do you read each time?

A. 5 B. 4 C. 3 D. 2 E. 1

22. How much can you understand when you read the reading part in Senior High School Entrance Examination or College Entrance Examination?

A. 80%~100% B. 60%~80% C. 50%~60% D. 30%~50% E. less than 30%

23. What about the ranking of your English grade in your class? Take the most recent written examination or College Entrance Examination as an example.

A. first five B. five to ten C. ten to twenty D. twenty to thirty E. after thirty

24. Your English test score of the SENIOR HIGH SCHOOL ENTRANCE EXAMINATION is: __________;

The full score of the English test of the SENIOR HIGH SCHOOL ENTRANCE EXAMINATION is: __________.

（**Items 25-37 are part of English proficiency test for UNIVERSITY STUDETS ONLY, please fill in the blanks or choose the correct answer to each.**）

25. Your English test score of the COLLEGE ENTRANCE EXAMINATION is:

Your written test score: __________; the written test full score: __________;

Your oral test score: __________.

26. 他关心你的前途。

A. He is concerned your future.

B. He concerns your future.

C. Your future concerns him.

D. Your future concerns about him.

27.小明是一个勤奋的学生，相反地，小玲非常懒惰。

A. Xiaoming is a diligent student, on the contrary Xiaoling is very lazy.

B. Xiaoming is a diligent student while Xiaoling is very lazy.

C. Xiaoming is a diligent student as Xiaoling is very lazy.

D. Xiaoming is a diligent student. In contrast, Xiaoling is very lazy.

28. 我很难拒绝她的要求。

A. I difficult reject her request.

B. I find it difficultly to reject her request.

C. It is difficult for me to reject her request.

D. I reject her request difficultly.

29. 图书馆让我把书还回去。

A. The library asked me to return the book back.

B. The library asked me to return back the book.

C. The library asked me to return the book.

D. I was asked to return back the book by the library.

30. 这次练习向学生们展示学习英语很有趣。

A. This exercise is to show students that learning English is fun.

B. This exercise is to show that students' learning English is fun.

C. This exercise is to show that learning English is fun.

D. This is exercise is to show students that learning English is fun.

31. _________ the Municipal government, I wish to extend our warm welcome to the friends who have come to visit our city.

A. On behalf of

B. On the behalf of

C. On behalf

D. Behalf of

32. Although Wild Aid has been trying to stop the slaughter of sharks for their fins, current regulations rarely curtail (缩减) _________ to the degree needed to restore shark population.

A. sharks are hunted

B. the hunting of sharks

C. to hunt sharks

D. sharks hunted

33. Post-consumer anxiety is the type of psychological tension _________ after making a major buying decision.

A. a person who may feel

B. and a person may feel

C. a person may feel

D. may feel a person

34. Although pecans are most plentiful in the southeastern part of the United States, they are found __________ Ohio and Illinois.

A. far north

B. north as far

C. as far north as

D. farthest north

35. Geologists __________ the large removal of pumice in volcanic areas like Mount St. Helens, because they know it is so important to revitalize the soil.

A. increasingly are concerned with

B. are concerned with increasingly

C. are increasingly concerned with

D. increasingly are concerning with

36. When two words are joined to make a new special meaning, its __________ and the result is called a compound.

A. meaning joins up

B. meaning joining up

C. joining up of the meaning

D. joined up meaning

37. Sunlight during dawn or dusk comes in at a lower angle, and since it cannot escape the dust and population of the atmosphere __________, the sky turns orange and then red.

A. so with daytime sunlight can

B. so can daytime sunlight

C. as with daytime sunlight can

D. as can the daytime sunlight
