# Supplementary material for: How Does a Healthy Interactive Environment Sustain Foreign Language Development? An Ecocontextualized Approach
Source: Int J Environ Res Public Health. 2022 Aug 19;19(16):10342. doi: 10.3390/ijerph191610342 (PMC9408107; doi:10.3390/ijerph191610342)
Supplement: Supplementary file 1 [file ijerph-19-10342-s001.zip › S2.pdf]

Supplemental material S2

Interview Questions

1. When and how did you start learning English? Did you start with English letters---ABC?
2. What English films or cartoons impressed you most? Why?
3. What is your opinion on College Entrance Examination in China?
4. What is your favorite subject? Why do you like it so much?
